# In Vivo Bone Tissue Engineering Strategies: Advances and Prospects

**DOI:** 10.3390/polym14153222

**Published:** 2022-08-08

**Authors:** Ilya L. Tsiklin, Aleksey V. Shabunin, Alexandr V. Kolsanov, Larisa T. Volova

**Affiliations:** 1 Biotechnology Center “Biotech”, Samara State Medical University, 443079 Samara, Russia; 2 City Clinical Hospital Botkin, Moscow Healthcare Department, 125284 Moscow, Russia

**Keywords:** bone tissue engineering, bone regeneration, scaffold, stem cells, growth factors, bioreactor, axial vascularization, flap prefabrication, in vivo bioreactor

## Abstract

Reconstruction of critical-sized bone defects remains a tremendous challenge for surgeons worldwide. Despite the variety of surgical techniques, current clinical strategies for bone defect repair demonstrate significant limitations and drawbacks, including donor-site morbidity, poor anatomical match, insufficient bone volume, bone graft resorption, and rejection. Bone tissue engineering (BTE) has emerged as a novel approach to guided bone tissue regeneration. BTE focuses on in vitro manipulations with seed cells, growth factors and bioactive scaffolds using bioreactors. The successful clinical translation of BTE requires overcoming a number of significant challenges. Currently, insufficient vascularization is the critical limitation for viability of the bone tissue-engineered construct. Furthermore, efficacy and safety of the scaffolds cell-seeding and exogenous growth factors administration are still controversial. The in vivo bioreactor principle (IVB) is an exceptionally promising concept for the in vivo bone tissue regeneration in a predictable patient-specific manner. This concept is based on the self-regenerative capacity of the human body, and combines flap prefabrication and axial vascularization strategies. Multiple experimental studies on in vivo BTE strategies presented in this review demonstrate the efficacy of this approach. Routine clinical application of the in vivo bioreactor principle is the future direction of BTE; however, it requires further investigation for overcoming some significant limitations.

## 1. Introduction

Critical-sized bone defects reconstruction remains a tremendous challenge for surgeons and a burden for the healthcare system worldwide. These defects cause considerable functional and cosmetic disorders, and negatively impact the quality of life [1,2]. Despite the huge armamentarium of surgical techniques, current bone repair strategies demonstrate significant limitations.

Among the ample variety of methods, the use of autologous bone grafts is the gold standard for bone defects reconstruction. Bone autografts demonstrate osteoinductive and osteoconductive properties due to their containing growth factors and recruitment of stem cells, however, their usage concerns donor-site morbidity, poor anatomical match, insufficient bone volume, and bone graft resorption [3,4].

Although vascularized bone flaps (e.g., fibula, scapula, or iliac crest) show a predictably high survival rate due to reliable blood supply, their harvesting can result in significant donor-site morbidity including chronic pain, lameness, hernia, ankle instability, etc. Moreover, such complications, as complete or partial flap failure, postoperative fistula, dehiscence, and bone exposure can occur [5,6,7]. Free flap harvest and revascularization substantially increase intraoperative blood loss volume, complexity, and duration of surgeries [8].

Allogeneic and xenogeneic bone graft materials are the most commonly-used alternatives to autologous bone. Along with off-the-shelf availability, human-derived or deproteinized bovine-derived bone substitutes have a long shelf life [9].

The development of computer 3D planning and prototyping methods contributed to the clinical application of patient-specific titanium and synthetic implants for extensive bone defects reconstructions [10,11]. Despite high-precision manufacturing and optimal anatomical match of the implants, the complication rate is significant. Complications predominantly include fractures, instability, extrusion, and rejection of implants. Furthermore, custom-made implants are associated with consequential financial costs [12,13,14].

Distraction osteogenesis has become particularly popular in treating patients with long bones defects; however, it is technically challenging and not widely used in craniofacial bone reconstruction due to the complex three-dimensional configuration of the defects and high surgical complications rate [15,16].

Therefore, it is of critical need for improved bone defects reconstruction methods nowadays. Current trends in bone reconstructive surgery include reducing functional donor-site morbidity, overcoming bone graft volume limitations, and improving the geometrical match of the graft for the recipient site. Bone tissue engineering (BTE) has emerged as a novel approach to bone defects repair and regeneration. This approach is based on in vitro manipulation of seed cells, growth factors and bioactive scaffolds using various bioreactors. The application of BTE is one of the promising trends for researchers globally due to recent advances in the development of various biocompatible scaffolds and cell technologies [17,18,19,20]. Currently, in vitro and in vivo experimental studies on BTE are widely presented, however, the routine clinical application is associated with certain limitations.

This study aims to extensively review research papers related to recent developments in BTE including advances and prospects of the in vivo BTE strategies.

## 2. Recent Developments in Bone Tissue Engineering

**Figure 1:**
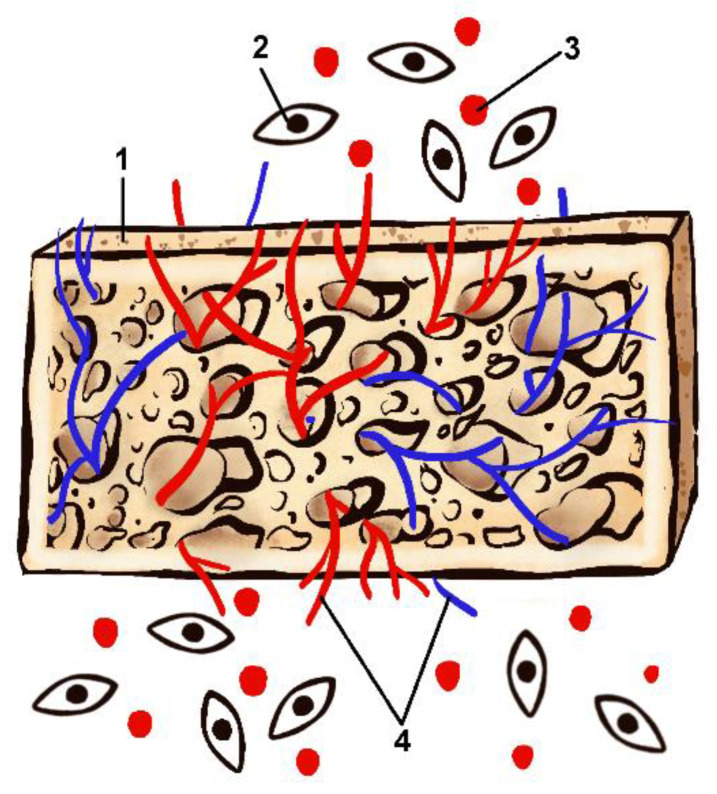
Key components of bone tissue regeneration (1-scaffold; 2-osteoprogenitor cells; 3-growth factors; 4-vascular network).

Previous studies have revealed key components of BTE [21,22,23,24,25,26]. According to these studies successful bone regeneration relies on the combination of (1) a bioactive scaffold [27,28,29,30,31]; (2) osteoprogenitor cells [21,26,32,33,34,35]; (3) growth factors (GF) [23,36,37,38,39,40] and (4) adequate vascularization [41,42,43] (Figure 1).

### 2.1. Scaffolds in Bone Tissue Engineering

A scaffold is an essential component of the bone tissue-engineered construct. It is a three-dimensional matrix with a porous microstructure imitating the extracellular matrix (ECM). Scaffolds with essential physical, chemical and biological properties seek to create appropriate regenerative microenvironment by providing conditions for cell adhesion, proliferation, angiogenesis, and the transport of GF. While discussing the crucial properties of a scaffold for bone tissue regeneration, it is mandatory to highlight: (1) biocompatibility, or biomimetic properties [28,44,45], (2) mechanical properties [22,46] and (3) biodegradability [47,48]. Some authors among the important requirements for an ideal scaffold emphasize the porosity and pore size [49], cell attachment properties [46,50], ability to visualize, histologically investigate, and sterilize the tissue-engineered construct [51,52,53,54].

Commonly used groups of materials for the fabrication of scaffolds include natural and synthetic polymers (including hydrogels), and bioceramics [26,52]. Additionally, many authors highlight the efficiency of metallic, biological, and composite scaffolds for BTE [55,56,57,58,59,60,61]. A list of scaffold fabrication techniques includes lyophilization, freeze and solvent casting, gas and microfluidic foaming, melt and compression molding, particulate leaching, phase separation process, and electrospinning [62,63,64]. Significant progress in manufacturing scaffolds has emerged due to the development and implementation of 3D printing technology [65,66]. Additive manufacturing approaches are classified in to extrusion-based, powder-based, and photopolymerization techniques [62,67,68,69]. The main types of the scaffolds for BTE, their crucial properties, fabrication and sterilization techniques are summarized in Table 1.

#### 2.1.1. Natural Polymers

Natural polymers are particularly effective for regenerative medicine application for two main reasons. Firstly, their structure is similar to the components of the ECM. Such a similarity enhances the osteoinductive and osteoconductive properties of the scaffolds. Secondly, the ability of natural polymers to swell significantly in the moist microenvironment allows them to simulate the hydrated state of living tissues [70,71,72]. This group of polymers includes collagen, chitosan, hyaluronic acid, fibrin and cellulose. Natural polymeric scaffolds can be created via electrospinning, lyophilization, salt-leaching, and 3D printing (fused deposition modeling).

In the past decades due to the *collagen* structure and function investigation, various collagen-based scaffolds have been proposed for BTE applications [73,74,75]. Collagen, as a connective tissue fibrillar protein, demonstrates biomimetic properties, high elasticity, and provides appropriate conditions for cells adhesion and interaction [76]. Along with hydroxyapatite it forms the basis of the bone tissue [77]. However, low mechanical strength and high biodegradability rate are considerable drawbacks of collagen as a scaffold for BTE [78]. Furthermore, short non-triple helical telopeptides in the collagen structure demonstrate immunogenicity [79,80].

Another natural scaffold material for tissue engineering purposes is *chitosan*. This biodegradable polymer can be found in the cuticle of insects, the cell wall of fungi and the shell crusts of crustaceans [81,82]. Chitosan shows excellent biocompatibility, low immunogenicity, and antimicrobial properties against certain fungi and bacteria. The main disadvantage of chitosan is its low mechanical strength, which can be overcome by means of combining this polymer with other materials [81,83].

*Hyaluronic acid*, as an important component of the ECM, has been widely used in bone regeneration, in particular in the craniofacial and alveolar bone reconstruction [84,85]. This natural polymer can be effectively used as a cell-seeded scaffold or a carrier for bioactive components due to its elasticity, flexibility, and biocompatibility. Furthermore, hyaluronic acid promotes and accelerates angiogenesis and cell migration [84,85].

*Fibrin* is a natural biopolymer derived from human blood. It is formed in the last step of the clotting cascade by the action of thrombin on fibrinogen. It demonstrates unique viscoelastic properties [23]. Mechanical stress causes irreversible deformation of the fibrin network, but it completely recovers its stiffness after removing the stress. Due to the poor rigidity of fibrin, it is commonly used for coating on metals, polymers, bioceramics, and other materials with stable mechanical properties. Moreover, coating on scaffolds noticeably enhances cell adhesion, proliferation, vasculogenesis, and, consequently, bone regeneration [86].

*Cellulose* is an organic compound with a polysaccharide structure. It can be derived from wooden and non-wooden plants, and some kind of bacteria [87]. Bacterial, fibrillated and crystalline types of cellulose have been studied on their potential in tissue engineering applications due to their physico-chemical properties, biocompatibility and biodegradability. Although mechanical rigidity of the cellulose is lacking, it can be used as the component of the composite scaffold. Murizan et al. reported the capability of nanocrystalline cellulose to enhance mechanical properties, tensile and compression of bone scaffolds [87].

#### 2.1.2. Synthetic Polymers

Synthetic polymers are widely used in BTE, and include biodegradable (polylactic acid—PLA, Polycaprolactone—PCL, polylactic-co-glycolic acid—PLGA), and non-biodegradable (Polyethylene Glycol—PEG, Polyurethane—PU, polyvinyl alcohol—PVA, poly 2-hydroxyethyl methacrylate—pHEMA) scaffolds [88,89,90,91]. Synthetic polymer fabrication is generally performed using salt-leaching, 3D printing, fused deposition modeling, and stereolithography techniques. Biodegradable synthetic polymers demonstrate biocompatibility, biodegradability, and controlled degradation rate [81,88,92]. PLA is the most commonly used polymer for tissue engineering applications. Both L and D forms of polylactide demonstrate high crystallinity and have identical melting temperature. Poly(L-lactide) shows low biodegradation rate, while poly(L/D-lactide), as an amorphous material, demonstrates high biodegradation rate and poor mechanical properties [93]. Thus, combining Poly(L-lactide) and poly(L/D-lactide) allows to achieve optimal biodegradation rate and enhances mechanical properties of the scaffold [94]. Polycaprolactone (PCL) has been widely investigated for BTE applications, however, it demonstrates poor cell adhesion and low mechanical properties [95]. PCL is commonly used as a component for composite scaffolds [96,97,98]. Another widely used synthetic polymer for tissue engineering applications is PLGA. Similarly to the above mentioned polymers, due to its low mechanical strength and rigidity, PLGA often plays the role of a biocompatible component, included in a composite scaffold based on substances with appropriate mechanical stability (hydroxyapatite, beta-tricalcium phosphate, TiO_2_) [99,100,101].

Although PEG, PU, PVA, pHEMA and other non-biodegradable synthetic polymers show excellent biocompatibility and appropriate flexibility, their use for BTE purposes is significantly limited by their low mechanical stability, poor cell attachment property, and lack of biodegradability [102,103].

Recent bone and cartilage tissue engineering studies have focused on hydrogels as highly swollen and porous 3 dimensional polymer networks that are able to keep moist microenvironment and to absorb inflammation exudate [104]. Furthermore, hydrogels demonstrate osteoconductivity, significant potential in the delivery of cells, nutrients, and GF [26,105,106]. Despite hydrophilic characteristic, good biocompatibility, and porosity, some authors report the need for the structural and mechanical property improvement of hydrogels [107,108]. Mechanical integrity can be improved through increasing crosslink density or creating more complex hydrogel systems with multiple polymers [107]. Another challenge occurs in using hydrogels in the oral cavity environment, where they are exposed to saliva [109].

#### 2.1.3. Bioceramic and Bioglass Scaffolds

Bioceramic scaffolds show excellent biomimetic and mechanical properties. They can be easily sterilized and visualized throughout the process of regeneration. Multiple fabrication techniques can be used to create bioceramic scaffolds, including particle and salt leaching, gas foaming, phase separation, and additive manufacturing techniques (selective laser sintering, fused deposition modeling, stereolithography and electron beam melting).

*Hydroxyapatite* (*HA*), is the key mineral component of the bone tissue with an interconnected porous and isotropic structure [110]. HA is excellent for bone tissue regeneration due to high biocompatibility, osteoconductive and osteoinductive properties [111,112]. Thus, Liu et al. demonstrated a significantly high volume and trabecular number of the newly formed bone using porcine HA for alveolar ridge guided bone regeneration [112]. However, due to the brittle structure of the HA, it is typically used in combination with other components to enhance their osteoinductive and osteoconductive properties [113,114].

*Tricalcium phosphate* (TCP) is a tertiary calcium phosphate, and it is widely used in scaffold-based BTE [115,116,117]. TCP is a biodegradable material rich with phosphorus and calcium, and it is commonly used in combination with polymers as a composite scaffold [118], or drug-loaded material [119]. However, low biodegradability rate is considered as a drawback of TCP scaffolds. Along with HA and TCP *Bioglass* demonstrates mechanical stability and biomimetic properties [89].

#### 2.1.4. Metallic Scaffolds

Nowadays, the application of bioabsorbable metallic scaffolds for BTE is of great interest for various researchers, due to recent advances in their structure and properties studies, and manufacturing development [58,59,112]. The use of zinc (Zn), magnesium (Mg), iron (Fe) and their alloys are increasingly gaining popularity for BTE applications. The porous Zn scaffolds demonstrate adequate mechanical properties matching those of trabecular bone, as well as having interconnected pore structure that can enhance cell adhesion and bone tissue ingrowth. Moreover, these scaffolds possess suitable biodegradation rates to allow for simultaneous new bone formation [59]. Among biocompatible materials for bone defects repair metals demonstrate the highest mechanical strength and stiffness, osteoconductive properties and ability to promote osteointegration. Metal stiffness is increasing during the early stages of the corrosion process [120]. Strength and stiffness are particularly important for load-bearing applications of the metallic constructs. Furthermore, powder-based fabrication techniques enable to create individualized metallic scaffolds [120,121].

#### 2.1.5. Biologic Scaffolds

The use of the biologic scaffold materials for BTE applications is based on the concept of cell removal and preserving the structural and functional components of the bone ECM [60]. Therefore, the main goal of such approach is recellularization and remodeling of the bone tissue structure. Xenogeneic decellularized bone is typically bovine-derived. Allogeneic scaffolds come from different human bones. Physical decellularization includes freeze–thaw and osmotic pressure procedures that cause cell lysis without critical disruption of the organic structure of the tissue. During freezing and thawing ice crystals penetrate cell membranes, while hypertonic or hypotonic solutions can disrupt the plasma membrane through osmotic shock. Other physical decellularization approaches include the use of hydrostatic pressure, ultrasound and electroporation [122]. Traditionally, lyophilization (freeze-drying technique) is used for decellularized bone scaffolds fabrication [61,123]. The most common sterilization method for decellularized bone is irradiation, however, the combination of ethanol, ultraviolet, or antibiotic can be also used for sterilization and disinfection [124]. Microstructure of the bone ECM is identical to the native one and provides optimal conditions of the cell recruitment, adhesion and proliferation. Furthermore, biologic scaffolds demonstrate significant osteogenic properties. We have recently reported the results of the microstructural and biochemical analysis of the lyophilized allogeneic human spongiosa [125]. Microstructural analysis revealed the hierarchical porous structure of the graft with complete removal of the cellular debris and bone marrow components. Moreover, the proteomic analysis confirmed the preservation of the extracellular matrix organic structure, including collagen and extracellular proteins, stimulating and inhibiting cell adhesion, proliferation, and differentiation [125]. However, some authors reported on the possibility of the foreign body response, encapsulation, and proinflammatory macrophage activation as a result of using xenogeneic ECM. Overall, the use of the biologic scaffolds is a perspective alternative to other groups of scaffolds, which provides solutions unavailable with the use of the synthetic scaffolds [60].

#### 2.1.6. Composite Scaffolds

Over the past decade, numerous efforts to create an ideal scaffold for BTE have been focused on combining various materials that provide biocompatibilty, biodegradability and adequate mechanical properties [44,56,83,123].

Most commonly bioceramics are combined with natural polymers, e.g., chitosan, collagen [56,57,118,126]. The main purpose of such a combination is to promote the new bone tissue formation, and to enhance the compressive strength of the scaffold. Thus, Xing et al. demonstrated enhanced osteoconductive properties of the mechanically rigid scaffold composed of chitin, collagen and hydroxyapatite [57].

Some authors successfully applied composite scaffolds incorporating graphene [27,55,127], and silk [127,128]. Kang et al. presented an experimental study on the combination of chitosan and allogeneic bone powder. This composite scaffold demonstrated suitable porosity and osteogenic properties [123]. Natarajan et al. presented the group of rare earth metal-based nanoparticles (cerium, gold, europium, gadolinium and others) as emerging perspective biomaterials for BTE. According to the study results these materials provide unique biocompatibility, osteogenic, antimicrobial, antioxidant, angiogenic, immunomodulatory, and anti-inflammatory properties [129].

While analyzing the application of different scaffolds for BTE, the presented literature review demonstrates the tendency to combine various biocompatible materials with appropriate properties [27,44,118,129,130,131]. Despite the above-mentioned variety of biomimetic, biodegradable, and mechanically stable materials, scientists continue efforts to create an ideal scaffold for BTE purposes.

### 2.2. Cellular Approach in BTE

The cellular approach in BTE is based on preliminary transplantation of autologous stem cells onto the scaffolds surface followed by their implantation into the defect site. Autologous multipotent mesenchymal stem cells (MMSCs) have been traditionally used for cell-loading procedures [35,44,132,133]. MMSCs demonstrate great perspective in cell-based tissue regeneration due to their proliferation, differentiation, and multilineage potential, as well as immune regulatory, and anti-inflammatory effects [134]. The essential criteria for MMSCs use in regenerative medicine include: (1) the ability to adhesion during in vitro cultivation; (2) expression of important specific antigens (SH-2, SH-3, SH-4, STRO-1, CD44, CD29, CD71, CD106, CD120a, CD124), (3) high proliferation potential (easily induced osteogenic, adipogenic, chondrogenic, neurogenic, and myogenic differentiation) [135]. Such bioactive substances as ascorbic acid, β glycerophosphate, dihydroxyvitamin D3, steroid hormones, and bone morphogenetic proteins can induce osteogenic differentiation of MMSCs. The main sources of MMSCs and their differentiation potential are presented in Table 2.

A number of studies have shown that transplanting MMSCs in vivo can increase the healing of damaged tissue [34,44,133,136,137]. Their homing, migration, proliferation, and differentiation are regulated by chemical and mechanical factors. Thus, osteopontin and stromal derived factor-1 (SDF-1) increase MMSCs migration and survival ability [138,139]. Different GF (basic fibroblast growth factor—bFGF, vascular endothelial growth factor—VEGF, insulin-like growth factor-1—IGF-1, platelet-derived growth factor—PDGF, transforming growth factor β1—TGF-β1, and others) are critical for the process of MMSCs homing and inducing tissue regeneration [136]. MMSCs differentiation significantly depends on the bioactive scaffold material and its mechanical properties—roughness, rigidity, microarchitectonics, and pore size [50,140].

Despite the advantages presented above, current concepts on the in vivo behavior of MMSCs, including differentiation and survival potential, immunomodulatory functions, tumorigenicity, and paracrine effects are still controversial [132,141,142]. Moreover, the type of cells to be seeded on the scaffolds (MMSCs, progenitor cells, or differentiated cells), and optimal cell culture conditions are still debated [35,132,134,136,141,142]. Lazennec et al. reported the ability of MMSCs to home to tumor sites with further tumor growth suppression or stimulation [141]. Molecular studies presented by Gunn et al. have demonstrated the role of IL-6 secreted by MSCs in proliferation and progression of multiple myeloma [142]. Current research on MMSCs focuses on further preclinical and clinical investigations of their behavior, safety, and therapeutic efficacy in vivo.

### 2.3. Growth Factors in Bone Tissue Engineering

Various GFs play crucial roles in bone tissue regeneration [22,38,39,40,143]. Osteoinductive GFs such as bone morphogenetic proteins (BMPs), vascular endothelial growth factors (VEGFs), platelet-derived growth factors (PDGFs), insulin-like growth factors (IGFs), transforming growth factors (TGFs-ß), fibroblast growth factors (FGF) promote bone tissue and vascular growth, regulate cell behavior, including recruitment, migration, adhesion, proliferation, and differentiation. Osteogenic GF have been widely used for BTE purposes. BMPs have gained particular popularity due to their well-known ability to promote cell migration, osteogenic differentiation of MSCs, and bone tissue formation [144,145]. The controlled delivery of GF within bioactive scaffolds enhances osteoprogenitor bone cells migration, proliferation, and differentiation, stimulates angiogenesis and, as a result, functional bone tissue regeneration in vitro and in vivo [146].

Jacinto-Tinajero et al. for the first time demonstrated efficacy of the plant-based GF for ectopic bone formation. Such GF as BMP-2, BMP-7 and TGF-β1 were produced in tobacco leaves in high amounts and were successfully used for BTE purposes [147].

Over the past decades, platelet-rich plasma (PRP) and platelet-rich fibrin (PRF) have been proactively applied in bone augmentation procedures as a natural source of multiple GF (INF-γ, TNF-α, MCP-1, MIP-1a, RANTES, bFGF, PDGF, VEGF), cytokines (IL-1b, IL-1ra, IL-4, IL-6, IL-8, IL-12, IL-13, IL-17, IL-2, IL-5, IL-7, IL-9, IL-10, IL-15), and chemokines (G-CSF, GM-CSF, Eotaxin, CXCL10 chemokine (IP-10), MIP 1b) that hypothetically improve bone tissue regeneration [40,148,149]. Combination of such platelet concentrates with natural and synthetic biomaterials can significantly enhance their effectiveness in bone tissue regeneration [40]. The main groups of GFs, their sources and functions are summarized in Table 3.

Despite the crucial role of GFs in bone tissue regeneration, adverse effects of their use are widely presented. Thus, Tannoury et al. reported complications with the use of recombinant human BMP 2 (rhBMP-2). Authors presented a wide spectrum of adverse outcomes related to rhBMP-2 use in spine surgeries, including ectopic bone formation, antibodies formation, vertebral osteolysis, wound healing complications, hematoma formation, as well as hypothetical tumorigenicity concerns [150].

### 2.4. In Vitro Vascularization Strategies in Bone Tissue Engineering

Vascularization is a fundamental requirement for the viability of the bone tissue-engineered construct. Development of the predictably sufficient vascular network within the construct remains one of the main challenges of BTE [43,151,152,153,154]. A stable blood supply to the bone scaffold provides an influx of oxygen, nutrients, GF, and osteoprogenitor cells, that are essential for bone tissue regeneration and remodeling. Therefore, the lack of the scaffold vascularization leads to oxygen and nutrients deficiency, accumulation of waste products, and, consequently, graft failure [155]. Insufficient vascularization is a considerable limit for the creation of large-sized tissue-engineered bone constructs. Various strategies for in vitro vascularization of the bone tissue constructs have been developed and implemented in the past. Vascular network creation can be achieved by means of several approaches: (1) use of the angiogenic GF, (2) use of the angiogenic cell cultures, (3) hypoxia-induced vascularization, (4) use of microvascular adipose tissue fragments, (5) 3D-bioprinting. Furthermore, mechanical stimulation, microfabrication and microfluidic techniques can be used as vascularization strategies [156,157]. Basic in vitro tissue vascularization strategies are presented in Table 4.

Kazimierczak et al. recommend to seed the scaffold with various types of cells for creating a functional vascularized bone graft in vitro. Authors used co-cultures of mesenchymal stem cells with endothelial cells to produce such a graft [158]. Currently, bioprinting-based vascularization strategy represents the most advanced technology of vascular network creation. 3D bioprinting of the vascular network is a promising approach, however, further preclinical studies are required [159]. Regardless of in vitro vascularization strategies variety, their use is characterized by a low vascular network formation rate. Therefore, poor in vitro vascularization of the bone tissue engineered construct is one of the considerable limits for routine BTE clinical application [155].

### 2.5. Bioreactors for In Vitro Bone Tissue Engineering

Bioreactors have been developed as essential devices for in vitro tissue engineering purposes to provide physiological tissue-specific environment by mimicking in vivo conditions for tissue growth and regeneration [158,160,161,162]. Various types of bioreactors for tissue engineering have been developed and applied in recent years.

*Perfusion bioreactors* use a pump system to provide continuous or non-continuous media perfusion through the cell-seeded scaffolds with interconnective porous structure and to enhance cell distribution and synthesis of the ECM. Perfusion bioreactors consist of a media reservoir, a pump, a flow perfusion chambers, an oxygenator, a tubing circuit, and a waste tank. Several authors reported successful use of perfusion bioreactors for BTE purposes [163,164,165]. Liu et al. presented results of using porcine decellularized native bone seeded with human smooth muscle cells and human umbilical vein endothelial cells within a perfusion bioreactor. Authors demonstrated improved density of cells and increased vascularization [163]. Ressler et al. confirmed osteogenic differentiation using a composite Calcium phosphate/Hydroxyapatite scaffold seeded with human mesenchymal stem cells in a perfusion bioreactor [164]. Pereira et al. used a custom-made perfusion bioreactor system and decellularized human bone scaffolds seeded with human bone marrow-derived mesenchymal stem cells [165].

*Spinner Flask Bioreactors* are composed of a media reservoir with two side arms with filter caps for gas exchange. Such a device design provides a convective flow and produces hydrodynamic forces to enhance mass transport [158,162]. It can effectively mimic important aspects of native bone environment and can positively affect accelerating human mesenchymal stem cells osteogenic differentiation [162]. The calcium content, alkaline phosphatase activity, and osteopontin secretion can be used as indicators of mesenchymal stem cells differentiation [166].

*Rotating bioreactors* provide unique microgravity conditions for minimizing shear stress and unloading the gravitational force typically placed on cell cultures. These bioreactors are composed of two concentric cylinders: the outer cylinder incorporates the culture medium chamber for placing the scaffold, and the inner one provides gas exchange [162,167]. The influence of the simulated microgravity environment on mesenchymal cell proliferation and osteogenic differentiation is still debated. Some authors reported preservation of the human bone marrow stem cell’s ability to differentiate into osteoblasts [168,169], while others revealed inhibiting effect of microgravity conditions on mesenchymal cells osteogenic differentiation [170].

*Pulsed Electromagnetic Fields-Based Bioreactors* have been recently applied as an effective treatment option in orthopedic clinical practice to support bone healing in patients with non-union or delayed-union bone fractures [158,171]. Furthermore, pulsed electromagnetic fields demonstrated efficiency in osteonecrosis and diabetic osteopenia treatment [172], and accelerate migration and osteogenic differentiation of mesenchymal stem cells, as well as osteoblast proliferation and differentiation in vitro [173]. Additionally, it was found that pulsed electromagnetic fields decrease the level of proinflammatory cytokines and increase the expression of anti-inflammatory cytokines [158,174].

## 3. In Vivo Bone Tissue Engineering Advances

Although the above-presented critical components of BTE have been thoroughly investigated in recent decades, their ideal combination to provide predictable and controlled bone tissue regeneration process is still lacking. Current limitations of in vitro BTE, in particular poor vascularization, confirm the critical need for further development of this field. Flap prefabrication has emerged as a bridge between conventional reconstructive surgery and tissue-engineering. It is a promising strategy which allows to use precise customized flaps matching patient-specific needs [175]. Applied to bone reconstructive surgery flap prefabrication is a potential alternative to autologous bone graft harvest, that can significantly decrease donor-site morbidity.

### 3.1. Historical and Terminological Aspects of Flap Prefabrication

Historically, the term “prefabrication” first appeared in the record of house-building or manufacturing of ships and aircrafts. It initially means the assembling all the necessary components of a structure, and then transporting the assemblies to the site of construction [176]. The preliminary report first presented by Shen in 1981 included results of the experimental study and clinical application of the vascular pedicle implantation into the skin flap [177]. As a result, a skin flap of the desired location and design became axially vascularized. In 1994 Pribaz et al. introduced the concept of prevascularized flaps modification by implantation of tissue or other device into a flap prior to its local transposition or free distant transfer. Authors of this concept first suggested the term “prelamination” [178]. The most common example of flap prelamination is the rib cartilage subcutaneous implantation at the forearm followed by later harvest and free transfer of the prelaminated forearm composite skin-cartilage flap for ear reconstruction [179,180,181]. Tan (2004) used the term “vascular induction” to describe the phenomenon of an axial blood supply introduction to create new transplantable tissue [179].

### 3.2. In Vivo Bioreactor Approach to BTE: Experimental Studies

In 2005 Stevens et al. first introduced the term “in vivo bioreactor” (IVB) as a new concept for in vivo BTE. According to this concept large volumes of bone can be created in a predictable way, without the need for cell transplantation and GF administration. In an experimental study in rabbits, authors created a space between the surface of the long bone and the inner layer of the periosteum and filled this space with a biocompatible calcium-alginate gel. Radiographical and histological analysis of the bone harvested after a period of 6 weeks confirmed formation of the new bone tissue biomechanically identical to the native one. The authors emphasized the crucial role of the pluripotent cells of the periosteum in the bone regeneration process [182].

Another historically important experimental study on IVB approach was conducted by Holt et al. in 2005. This study on a rat model presented in vivo ectopic bone formation by combining an axial vascularization and prefabrication of the hydroxyapatite scaffold with a capability of further vascularized tissue transfer. Authors of this study demonstrated the creation of the rich vascular network inside the scaffold regardless of the administration of the BMP-2, however, supplementation of the BMP-2 could assumedly initiate pluripotent cells recruitment from circulating blood to new bone generation [183].

These two independent groundbreaking studies have become the starting point for further development of in vivo BTE strategies. Even though classical IVB, as it was underlined above, suggested the human body as the main source of progenitor cells and GF, many authors presented multiple modifications of the IVB by means of scaffolds cell-seeding and supplementation with exogenous GF [184,185,186,187,188,189,190]. IVB has been investigated in variety of small and large animal models [189,190,191,192,193,194,195,196]. Akar et al. presented basic requirements for IVB preclinical models, including the use of clinically translatable surgical techniques, choosing implantation sites with high regenerative potential and low infection risk, allowing quantitative evaluation of results, and availability in a wide range of research centers [191]. The vast majority of these experimental studies present the combination of the IVB critical components, which can be represented as a following formula.

IVB = S + FP + AV

(S—scaffold; FP—flap prefabrication, AV—axial vascularization).

According to the above-mentioned IVB formula it is logical to review and discuss this approach as an inseparable combination of axial vascularization and flap prefabrication methods. However, AV, as a separate component, may be optional if axially vascularized flap is used for scaffold prefabrication. Prior to discussion of the IVB strategies presented in literature in recent years, it is worth highlighting current tendencies in choosing scaffolds for in vivo BTE purposes.

#### 3.2.1. Scaffolds for In Vivo BTE

While reviewing multiple studies related to in vivo BTE, the spectrum of biocompatible materials most commonly used for in vivo BTE typically includes the bioceramic [184,185,187,191,197,198,199,200,201], allogeneic or xenogeneic bone-derived [189,193,194,195,202,203], and composite scaffolds [190,204,205]. This literature review evidently demonstrates the tendency to use mechanically stable materials with long controlled biodegradation rate for in vivo BTE purposes. Furthermore, some authors present efforts to improve properties of the scaffolds by combining various bioactive materials. Thus, Abu-Shahba used biohybrid bone blocks consisting of bovine-derived bone matrix in combination with PCL biodegradable polymer and collagen fragments for surface activation and scaffold reinforcement [204]. Kuzmenka et al. presented sol-gel hybrid glass scaffold integrated with calcium sources with the aim to create a bioactive implant with long-lasting calcium release while preserving its mechanical properties [206].

#### 3.2.2. In Vivo Vascularization and Prefabrication Strategies in BTE

Viability and growth support of the in vivo tissue engineered construct strictly depend on its constant and reliable blood supply. Despite the huge arsenal of in vitro angiogenesis methods, the lack of adequate vascularization remains the prevalent challenge and limitation in up-to-date BTE [201,207,208,209,210,211,212]. This fact resulted in the development of various in vivo vascularization strategies. Furthermore, local soft tissues at the bone defect site often have inappropriate quality and volume, and can be compromised due to cicatricial or post-radiation changes. According to this fact, ectopic bone graft prefabrication at the intact anatomic site can be an effective method to overcome such challenges.

**Figure 2:**
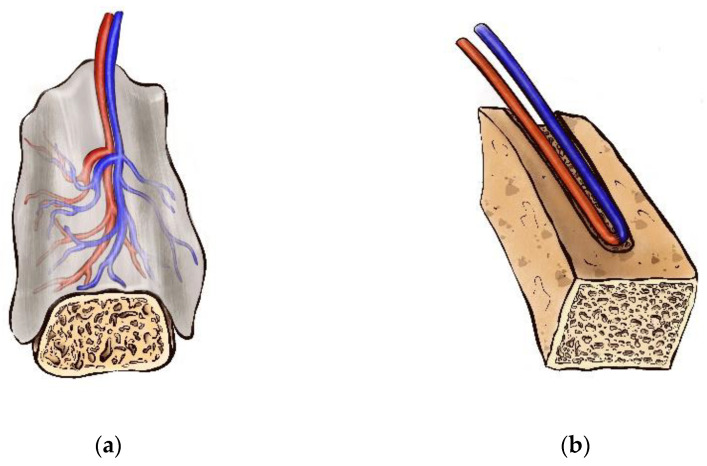
Scaffold axial vascularization strategy—(**a**) flap-based approach; (**b**) vessel-based approach.

The axial vascularization strategy has emerged as a powerful tool for rapid vascular network formation within a bioactive scaffold. This concept of vascularization has been expanded through the use of flap-based and vessel-based approaches [193,207] (Figure 2).

In the early seventies, the foundation of the axial pattern flaps revolutionized reconstructive surgery [213]. In contrast to random pattern flap approach, which predominantly relies on the angiogenesis within the host recipient site, anatomically stable arteriovenous system allows to induce predictable neo-vascularization of various scaffold biomaterials [207]. Thus, flap-based vascularization, that is also known as the “extrinsic” mode of neovascularization, can be classified in to random flap-based and axial flap-based types. While using random flaps, the neovascular bed originates from the periphery of the construct after its implantation into a highly vascularized environment [213]. According to this principle, subcutaneous, intramuscular, and intraperitoneal implantation have been reported [198,202,214]. At the same time, the presence of the stable arteriovenous axis provides constant and reliable blood supply of the flap, and progenitor cells and GF delivery. Furthermore, axially vascularized flap can be transferred to the defect site via its arteriovenous pedicle [207]. However, extrinsic vascularization demonstrates limited success for the vascular network creation in the central part of large scaffolds [215,216]. Kneser et al. described “intrinsic” vascularization mode as a tool for adequate perfusion of the large tissue-engineered construct and uniform vasculature distribution within its structure [217]. Extrinsic and intrinsic vascularization modes have been widely used in the experimental and clinical studies [202,216,217,218,219]. Various tissue types have been applied for bone grafts or scaffolds prefabrication in recent years, including subcutaneous pocket, periosteal, fascial, muscular, and omental flaps [147,193,198,204,214,220,221,222,223].

*Subcutaneous pocket* creation has gained popularity as a method of ectopic scaffold implantation due to simplicity of surgical technique, variety of potential prefabrication sites for random flap-based vascularization, and minimal donor-site morbidity [193]. This approach has been investigated by many authors in small and large animal models. Lee et.al created subcutaneous pockets in rats for cell-seeded and cell-free tibial condyle scaffolds implantation. The scaffolds were harvested and analyzed 6 weeks after implantation. Histomorphometry and immunohistochemical analysis demonstrated blood vessels and mineralized tissue formation within in vivo engineered bone grafts [215]. Wu et al. used cell-seeded synthetic b-TCP and coral-derived HA scaffolds for ectopic subcutaneous implantation at the back of Nude Mice. Twelve weeks after implantation, authors revealed the formation of the vascularized new bone tissue via histological analysis and micro-computed tomography [221]. Hypothetically, subcutaneously prefabricated bone flaps can be axially vascularized by means of anatomically consistent or transposed vascular pedicle if simultaneous bone and soft tissue repair is needed.

*Periosteal flaps* have been widely used for bone grafts prefabrication due to well-known high osteogenic potential and rich vasculature of the periosteum [191,193,194,198,204]. The inner cambium layer of the periosteum is well-vascularized and represents the rich source of clinically useful osteoprogenitor cells, including osteoblasts and multipotent stem cells. Despite the fact that osteoblasts may be absent in a cambial layer in adults, they can appear whenever required, for instance, for fracture healing [224]. The outer fibrous layer contains highly organized and directional collagen fibers (Sharpey’s fibers), and a smaller number of cells, mostly fibroblasts and pericytes [225,226]. As mentioned earlier, the first introduction of the IVB concept was based on the use of the periosteum cambial layer as the source of progenitor cells for bone regeneration [182]. Periosteal flaps have been predominantly investigated as an extrinsic approach to scaffolds vascularization and bone grafts prefabrication [193,198,227,228]. However, Sparks et al. suggested to use axially vascularized inverted corticoperiosteal flap. This approach provides an intrinsic axial blood supply, while the osteogenic surface of the periosteum can be located inside the scaffold [207]. Huang et al. presented a rabbit model of the bone graft prefabrication using a skull periosteal flap based on the supraorbital vessels, and confirmed new bone formation after 16 weeks of the graft prefabrication [193]. Ersoy used the combination of the periosto-fasciocutaneous flap transposed to the abdomen for bioactive glass and hydroxyapatite scaffolds prefabrication, and confirmed the osteogenic capacity of the vascularized periosteum [198]. Han et.al presented the preclinical model of the IVB in rabbits by combining β-TCP scaffold, tibial periosteal flap and the saphenous vascular pedicle, and confirmed the presence of the rich vascular network and new bone formation 4 weeks after prefabrication. New bone formation was mainly seen in peripheral aspects of the scaffold, while microvascular infusion and immunohistochemical staining showed direct revascularization of β-TCP scaffold [185]. Nau et al. used a rat model to evaluate the efficacy of periosteal flaps in bone defects healing. In various study groups, authors used periosteal flaps alone or in combination with β-TCP scaffold, bone marrow-derived mononuclear cells, and vascular pedicle. Prefabrication terms of four and eight weeks were analyzed. This strategy resulted in significantly improved bone healing [228]. Abu-Shahba et al. investigated the regenerative potential of the periosteal grafts and vascularized periosteal flaps in combination with muscle flaps in sheep model mandible defects reconstruction. The authors revealed enhanced new bone formation and enhanced vascularization after 13 and 23 weeks after alloplastic scaffolds implantation in both study groups by means of micro-CT and histological analysis [204].

Prelaminated *fascial flaps* have been successfully applied for complex tracheal, laryngeal and ear defects by many authors, by means of cartilage or alloplastic materials implantation underneath a radial forearm or temporoparietal fascia [180,229,230]. Prefabrication of bone grafts using fascial flaps has been also presented in series of preclinical studies. Fan et al. demonstrated efficacy of segmental bone defects repair in rhesus monkey using prevascularized cell-seeded scaffolds. These scaffolds were vascularized by saphenous arteriovenous bundle and covered with the fascial flap. Such vascularization and prefabrication approach resulted in new bone formation and capillary vessel in-growth [231]. Brey et al. conducted comparative analysis of periosteal and fascial flaps use for bone grafts prefabrication and vascularization. The analysis showed no significant difference in vascularization of the scaffolds and volume or shape of tissue formed. However, the use of the fascial flaps resulted in formation of predominantly fibrovascular tissue, while scaffolds that contacted with periosteal flaps demonstrated endochondral, direct, and appositional bone growth [232]. For clinical purposes random or axially vascularized fascial flaps for extrinsic vascularization of the scaffold can be harvested in a variety of anatomical locations, with minimal donor-site morbidity, however, osteogenic potential of fascia requires further investigations. Hypothetically, combination of fascial flaps with periosteal flaps and/or intrinsic vessel-based vascularization strategies can be considered as a logical future direction of the IVB concept development.

*Muscle flaps* have been effectively applied for in vivo BTE purposes [218,222,233,234]. It is well established that muscle tissue is a rich source of progenitor cells, including cells with osteogenic properties. Covering the fracture with muscle flaps provides a suitable environment for osteogenesis and reduces bone healing time [235].

Spalthoff et al. used prefabricated β-TCP scaffolds with or without a vascular bundle, combined with autologous bone marrow by implantation in the latissimus dorsi muscle in sheep. Histomorphometric analysis exhibited ectopic bone growth in all study groups with no significant difference between three- or six-months terms of prefabrication [234]. Kokemüller et al. reported experimental study on IVB approach based on prefabrication of the β-TCP scaffolds with a latissimus dorsi muscle flap and thoracodorsal vascular pedicle for axial construct perfusion in sheep. The authors confirmed considerably induced ectopic bone growth in all implanted scaffolds, and significantly increased bone growth, ceramic resorption and angiogenesis in scaffolds with axial perfusion [219]. Liu et al. presented IVB for vascularized bone graft engineering by implanting the composite bovine-derived scaffolds supplemented with recombinant human bone morphogenetic protein-7 (rhBMP-7) and/or recombinant human vascular endothelial growth factor 165 (rhVEGF165) in latissimus dorsi muscle in pigs. Histomorphometric analysis after twelve weeks of prefabrication revealed new lamellar and trabecular bone formation with higher bone density in scaffolds supplemented with rhVEGF165 [223]. Zhou et al. used demineralized freeze-dried bone allografts and coralline hydroxyapatite scaffolds with or without BMP stimulation, loaded into customized titanium meshes and prefabricated with latissimus dorsi muscle flaps in monkeys. Prefabricated bone grafts were used for mandible reconstruction thirteen weeks after implantation, and were observed in situ for another thirteen weeks. The authors analyzed the optimal time for prefabricated bone flap transfer via technetium-99m-methyl diphosphonate (Tc-MDP) single-photon emission computed tomography/computed tomography (SPECT/CT). According to the study results, authors suggested to transfer the flap at an interval of 8 to 13 weeks. At these terms the bone density gradually increased, while the uptake of 99 m Tc-MDP started to decrease from its peak at 8 weeks [236].

In addition to representing a rich source of progenitor cells, vasculogenic and osteogenic environment, vascularized muscle flaps can be found in a range of anatomic sites. In a clinical setting, latissimus dorsi, pectoralis, rectus abdominis, and rectus femoris muscles are widely used as free flaps due to having anatomically consistent vascular pedicles of an appropriate caliber and length.

*Omental flaps* have demonstrated osteogenic and vasculogenic potential for BTE purposes in multiple experimental studies [202,237,238]. Kamei et al. presented an experimental study in a rabbit model based on wrapping omentum with a periosteal graft followed by harvesting and analyzing omentum samples in 1, 2, 4, 6, 8, 12, or 24 weeks after surgery. Within 1 week after surgery, authors revealed the presence of osteoblasts clusters, while 8 weeks after prefabrication, medullization, including the presence of granulocytes, was observed [238]. Similarly, Sadegh et al. used free periosteal graft loaded with adipose tissue-derived stem cells for wrapping the pedicled omental flap in dog model. Such a tissue engineering approach lead to ectopic new bone formation [239]. Wiltfang et al. proposed the IVB strategy for ectopic bone formation based on the combination of titanium cages filled with bone mineral blocks, supplemented with recombinant human BMP-2, and bone marrow aspirate. These scaffolds were implanted into the gastrocolic omentum for a period of 3 months. Later, a free composite flap was harvested and transferred to the mandibular defect. Bone remodeling and mineralization both at the prefabrication and at the defect sites was confirmed by in vivo SPECT/CT [202]. Jacinto-Tinajero et al. presented the dog model of the IVB for BTE. In this study the scaffold consisted of collagen type 1 sponge, demineralized bone powder, calcium chloride, thrombin and PRP. The scaffold was wrapped with omentum and prefabricated for four months. As a result, a heterotopic trabecular bone formation was revealed [147]. Applied to clinical settings, omental flaps have been typically used for pharyngeal and esophageal defects reconstruction [239]. The feasibility of omental flaps clinical application for in vivo BTE purposes remains controversial and requires further experimental investigations.

While summarizing the efficacy of the flap-based techniques for in vivo BTE, it is mandatory to underline that the use of different tissue flaps for the scaffolds prefabrication demonstrates both osteogenic and vasculogenic capacity. Therefore, flap-based approach can be considered as an essential component of IVB structure. Axially vascularized flaps initiate bone tissue and blood vessels ingrowth within ectopically implanted scaffold and allow its consequent transposition or free transfer.

*Vessel-based axial vascularization* has revolutionized BTE due to the possibility to create rich and stable vascular network within a scaffold in a rapid and predictable manner [195,201,207,208,216,240,241,242]. As mentioned above, the presence of the vascular pedicle allows for the transposition of the flap locally, or to transfer it to the distant recipient site. Three main strategies of vessel-based axial vascularization include arteriovenous bundle (AVB), flow-through vascular bundle (FTVB), and arteriovenous loop (AV-loop).

*Arteriovenous bundle* (AVB) approach to tissue-engineered construct axial vascularization is based on the use of anatomically consistent vascular pedicle. It has emerged as a reliable method to provide a scaffold with a stable axial blood supply and constant perfusion. Moreover, AVB strategy allows to transfer the bone flap after a certain period of prefabrication using microsurgical techniques [186,241,243]. Polykandriotis et al. presented the experimental study on the use of AVB for BTE in rats. In this study the authors made an effort to create vascularized tissue-engineered construct suitable for microsurgical transfer. For this purpose, AVB was used as the source of an axial blood supply by inserting the pedicle into a specially designed channel in to the bovine cancellous bone-derived scaffold. The scaffold vascularization was evaluated at 2, 4, and 8 weeks after implantation by means of histological, histomorphometric analysis, scanning electron microscopy, and micro-magnetic resonance angiography for in vivo evaluation of the vascularized scaffolds. The vascularized constructs demonstrated well organized sprouting vascular network of high density and degree of maturation, with organization into vessels of different orders [242]. Li et al. used AVB for axial vascularization of the composite PLGA/ β-TCP scaffolds supplemented with rhBMP-2 in minipigs. The authors concluded that AVB significantly improved scaffold vascularization and new bone formation. Furthermore, defined vascular pedicle allows to transfer the flap to the bone defect by means of microvascular anastomoses [244].

*Flow-through vascular bundle* (FTVB) is an effective and relatively simple option for intrinsic vascularization of the scaffold. The main difference from the AVB approach is that the vascular pedicle is not ligated [195,244,245]. Yamaguchi et al. reported results of the vascularized allogeneic bone graft prefabrication in a rat model using flow-through saphenous bundle as a vascular carrier. Histological evaluations confirmed angiogenesis and bone formation in the group of axially vascularized scaffolds [195]. Yao et al. conducted an experimental study on IVB strategy in rabbits. Free radial bone grafts were harvested and vascularized by the external maxillary artery pedicle passed through the bone marrow cavity. India ink perfusion revealed high microvessel density comparing to the control group without axial blood supply. A peak of angiogenesis was indicated at four weeks postoperatively by means of integrated optical density of tetracycline fluorescence labelling. The authors concluded that increased angiogenesis enhanced osteogenesis in the tissue engineered construct [245].

*Arteriovenous loop* (AVL) has become the most commonly applied and perspective axial vascularization strategy described in multiple experimental studies on soft and bone tissue regeneration in recent decades [186,188,201,208,216,245,246,247]. The AVL approach to axial vascularization is based on the creation of the arteriovenous shunt with a purpose of rapid sprouting of the vascular network. This technique has been investigated in small and large animal models as the IVB approach to bone tissue regeneration. Kneser et al. reported the use of the AVL including artery, vein and vein graft, for bovine cancellous bone blocks axial vascularization in rats. Significant vascularization of the porous bone matrix occurred by the 8th week of observation and was confirmed by the intraaortic India ink perfusion [217]. Beier et al. presented results of the bovine cancellous bone block axial vascularization via AVL in a sheep model. AVL was microsurgically created in an isolation chamber. The tissue engineered constructs displayed increased axial vascularization, which was quantified by histomorphometric analysis and micro-computed tomography. In vivo sequential MRI demonstrated a significant progressive increase in the scaffold perfusion volume. Immunohistochemical analysis confirmed new blood vessels formation [248]. In 2011 Eweida et al. introduced the results of the cadaveric and surgical pilot studies in goats to test the potential model for axial vascularization of the mandible tissue engineered constructs. The aim of this study was to define the optimal vascular axis to create the AVL in the mandibular region. Facial artery and vein were considered as vessels of choice to vascularize scaffolds for mandible defects reconstruction. Later, in 2014, authors applied this model in vivo by means of successful facial artery—facial vein AVL creating in goats, and confirmed significantly enhanced vascularization in the central part of the scaffold comparing to non-vascularized scaffolds in the control group [243,249]. Ma et al. reported the rabbit model of the IVB for bone defects reconstruction. Authors used a combination of β-TCP scaffold, saphenous artery—saphenous vein AVL, and osteogenic cell sheet based on bone marrow-derived stem cells. The AVL was inserted into the specially prepared lateral grooves of the scaffold. The scaffold was then wrapped with the cell sheet. After eight weeks of prefabrication, histological and histomorphometric examinations revealed a formation of the trabecular bone tissue at the central part of the axially vascularized scaffold with the presence of osteoblasts, osteocytes, and bone marrow cavity-like structures surrounded by a dense matrix. The control group included scaffolds without axial blood supply, and displayed lamellar bone formation, predominantly at the edge of the construct, and sporadic bone formation in the central part of the scaffold [201].

### 3.3. In Vivo Bioreactor Approach to BTE: Clinical Application

Despite that numerous experimental studies on IVB in BTE using small and large animal models have confirmed efficiency of this strategy for ectopic bone prevascularization and regeneration, reports on the clinical application of this principle are still rare. We have reviewed clinical reports related to in vivo BTE and IVB principle presented in past decades. In 2016 Huang et al. summarized eight clinical reports on IVB approach to bone defects reconstruction between 1999 and 2014 [250]. The majority of the defects presented in this review included mandible defects repair using IVB principle and various prefabrication strategies [217,218,219,223,228,251,252]. In two of the eight mentioned cases, authors presented in situ tibial and radial bones defects repair, and for the first time used AVL in the clinical settings with excellent long-term results [220]. Additionally, the review of publications that could belong to the IVB approach to bone reconstruction for the same period of time revealed several more clinical cases.

Thus, in 2000, Safak et al. reported experimental studies on bone flap prefabrication, followed by two clinical cases of successful use of prefabricated iliac osteomyocutaneous flaps. The authors elevated pedicled split-inner cortex iliac bone flap and implanted it into the subcutaneous pocket in the medial groin region. After four weeks of prefabrication neovascularized composite flap was harvested and transferred to the defect based on the deep circumflex iliac vessels [253]. In 2001, Vacanti et al. presented a clinical report on reconstruction of an avulsed phalanx using in vivo BTE approach. As a result of the car accident, dorsal skin, nail bed, extensor tendon, and distal phalanx of the thumb had been lost. At the first stage the injured thumb was debrided and placed in to subcutaneous pocket at the abdomen for nineteen days. The skin flap healed successfully and was completely divided on nineteenth day. At the second stage porous HA scaffold was seeded with autologous radial periosteum-derived cells, incubated in vitro for nine weeks. Cell-seeded scaffold was implanted to the defect site twelve weeks after injury. MRI examination, conventional radiographs, and light-microscopical examination of the biopsy specimens, confirmed adequate vascularization of the scaffold and new lamellar bone formation within the tissue-engineered construct. However, histomorphometric analysis revealed that 5 percent of the construct incorporated lamellar bone and ossified endochondral tissue, while most of the volume was represented with soft tissue and blood vessels [254]. In 2004, Gronthos described a clinical case of the human mandible reconstruction using IVB approach. The author used prefabricated composite bone–muscle-flap. The scaffold used, consisted of a titanium mesh loaded with HA blocks seeded with bone marrow-derived stem cells, and supplemented by rhBMP-7. The tissue engineered construct was implanted into the latissimus dorsi muscle. After seven weeks of prefabrication the composite flap was transferred via microvascular anastomoses with the branches of external carotid artery and cephalic vein with good initial clinical results. During the follow up of the patient, a fracture of the titanium mesh, as well as partial necrosis of the bone flap were documented. The follow up was limited due to patient’s death from the cardiac arrest fifteen months after tissue engineered flap transfer [217,255,256]. In 2004 Yamada et al. successfully applied injectable tissue-engineered bone, based on bone marrow-derived stem cells and PRP, for maxilla and mandible augmentation in three partially edentulous patients [257]. In 2007 Marcacci et al. presented a new tissue engineering approach to surgical treatment of patients with extensive bone diaphysis defects. The authors applied porous HA ceramic scaffolds seeded with bone marrow-derived stem cells. Seven years follow-up demonstrated complete integration of the scaffold confirmed by conventional radiographs and computed tomography scans [258]. In 2008 Iino et al. reported the clinical use of the particulate cancellous bone and bone marrow, as an effective in vivo BTE approach to in situ maxillary and mandibular defects reconstruction, including patients with lip and palate cleft [259]. In 2009 Leonhardt et al. applied prefabricated radial forearm flaps for secondary mandible reconstruction in patients who had undergone tumor surgery. The authors harvested a cylinder of cancellous bone from the iliac crest, and implanted bone block in the forearm for prevascularization. After a period of 4 weeks the prefabricated flap was transferred into the mandibular defect [260]. In 2015 Sadigh et al. presented a case report on the anterolateral thigh flap prelamination with a fibula bone graft in a patient with the failure of the previously transferred free fibula flap. The fibula bone graft was inserted into a suprafascial pocket at the thigh for three weeks. After a period of prevascularization, the free transfer of the composite flap was successfully performed [261]. In 2016 Rüegg et al. presented a retrospective analysis of fifty clinical cases of facial bones defects reconstruction from 1988 to 2014 using prefabricated vascularized calvarium flaps. At the first stage of the procedure authors harvested calvarium bone flap and prelaminated it with a skin graft. After two to sixteen weeks of prefabrication the neovascularized composite flap was transferred to the facial bone defect. A long-term evaluation at the fifteen year follow-up demonstrated high success rate of such approach to bone defects reconstruction [262]. In 2019 Banaszewski et al. successfully used prefabricated corticoperiosteal medial femoral condyle free flap for one-stage laryngotracheal reconstruction [263].

Despite the variety of the IVB strategies used by the authors of the above-mentioned studies, the philosophy of the basic approach in all the studies relies on the effort to effectively combine the critical components of the bone tissue regeneration process.

## 4. Conclusions and Future Directions

Over the past decades, challenges in critical-sized bone defects repair inspired scientists and surgeons to develop new strategies for in vivo BTE, as innovative patient-specific solutions for minimally invasive bone defects reconstruction. The main efforts of these strategies aim to perform bone reconstructions using viable tissue-engineered constructs of precise anatomical shape and sufficient volume. Furthermore, minimal donor-site morbidity is one of the important criteria of in vivo BTE concept.

Although the essential components of the IVB have been thoroughly investigated in past years, their ideal combination is still debated. Thus, the ideal scaffold material is still a cause for discussion. Currently, the prevalent trend is to use crucial properties of various biocompatible scaffold materials through their combination.

Despite the advantages of the flap prefabrication technique, including prevascularization at the distant and intact ectopic anatomic site, the flap-based approach is associated with certain technical challenges, additional surgical site, and time required for scaffold initial integration. Moreover, according to the up-to-date literature, the terms of bone graft or scaffold prefabrication are still controversial.

The axial vascularization strategy is considered optimal for rapid vascular network spreading; however, it requires prefabrication of the grafts at the certain sites with anatomically consistent vascular pedicles. Furthermore, vessel-based axial vascularization, in particular AVL, requires complex microsurgical procedures, and therefore increases procedure duration and donor-site morbidity.

Further investigation of the safety and long-term effects of cell-seeding techniques and exogenous GF administration is required prior their routine clinical application. However, the self-regenerative capacity of the human body as the core principle of the IVB, along with overcoming the problem of insufficient vascularization of bone tissue-engineered construct seems to be an exceptionally promising approach to critical-sized bone defects repair, and can be recommended for further implication in clinical settings.

## Figures and Tables

**Table 1:** Scaffolds for bone tissue engineering.

Scaffold Type	Advantages	Disadvantages	Fabrication Technique	Sterilization Method
Natural polymers	Biocompatibility Cell adhesion, proliferation, Angiogenesis Low immunogenicity Antimicrobial properties	Poor mechanical strength High biodegradability rate	Electrospinning Lyophilization Salt-leaching 3D printing (fused deposition modeling)	Supercritical carbon dioxide Lyophilization combined with gas plasma Peracetic acid Ethanol UV irradiation
Synthetic polymers	Biocompatibility Appropriate mechanical stability Controlled biodegradation rate	Lack of degradation (in the group of non-biodegradable polymers)	Salt-leaching 3D printing (fused deposition modeling) Fused deposition modeling Stereolithography	Plasma sterilization (Hydrogen peroxide) Supercritical carbon dioxide Ethanol Antibiotics Dry heat Electron beam irradiation Gamma irradiation UV irradiation Ethylene oxide
Hydrogels	Biocompatibility Osteoconductivity, Cell adhesion, proliferation Hydrophilic characteristics Porosity	Poor mechanical strength	Electrospinning 3D printing	Ethanol Ethylene oxide Autoclaving Supercritical carbon dioxide Lyophilization Electron beam irradiation Gamma irradiation UV irradiation
Bioceramic scaffolds	Biocompatibility Porosity Osteoconductive and osteoinductive properties High mechanical strength Individualized scaffolds Easily sterilized and visualized Controlled pore size	Brittle structure (HA)	Particle/salt leaching Gas foaming Phase separation Selective laser sintering Fused deposition modeling Electron beam melting Stereolithography	Steam Dry heat Ethylene oxide Electron beam irradiation Gamma irradiation UV irradiation
Metallic scaffolds	Biocompatibility High mechanical strength and stiffness Osteoconductive properties Ability to promote osteointegration Individualized scaffolds Easily sterilized and visualized Controlled pore size	Corrosion	Stereolithography Electron beam melting Selective laser melting	Steam Dry heat Ethylene oxide (ETO) Electron beam irradiation Gamma irradiation UV irradiation
Biological scaffolds	Biomimetic properties Identical microstructure and porosity	Foreign body and inflammatory response	Lyophilization	Supercritical carbon dioxide Gamma irradiation
Composite scaffolds	Combination of different scaffolds advantages and compensating disadvantages	Combination of different scaffolds advantages and compensating disadvantages Limited new bone formation	Electrospinning Lyophilization Particle/salt leaching Gas foaming Phase separation Additive manufacturing techniques (selective laser sintering, fused deposition modeling and electron beam melting) Stereolithography	Electron beam irradiation Gamma irradiation UV irradiation

**Table 2:** Sources and differentiation potential of MMSCs.

Source of MMSCs	Differentiation Potential
Bone marrow MMSCs	Osteoblasts, Adipocytes, Myocytes, Neurons, Astrocytes, Hepatocytes, Cardiomyocytes, Chondrocytes, Mesangial cells
Adipose tissue MMSCs	Chondrocytes, Osteoblasts, Adipocytes, Myocytes
Tooth pulp MMSCs	Odontoblasts, Chondrocytes, Osteoblasts, Adipocytes
Muscle tissue MMSCs	Osteoblasts, Adipocytes, Chondrocytes, Neurons, Endothelial cells

**Table 3:** Growth factors in bone tissue engineering.

Growth Factors	Sources	Functions
Bone Morphogenic Protein (BMP)	MMSCs Osteoblasts Endothelial cells Chondrocytes	Induction of the bone growth Cells migration, proliferation, differentiation
Vascular Endothelial Growth Factor (VEGF)	Platelets Osteoblasts Chondrocytes Endothelial cells	Angiogenesis (regulation of migration and proliferation of endothelial cells)
Platelet-Derived Growth Factor (PDGF)	Platelets Osteoblasts Endothelial cells	Bone cells proliferation Angiogenesis
Transforming Growth Factor-Beta (TGF-β)	Platelets Osteoblasts Chondrocytes Endothelial cells Fibroblasts	Induction of the bone growth Osteoprogenitor cells migration, proliferation, differentiation
Fibroblast Growth Factor (FGF)	MMSCs Osteoblasts Chondrocytes Endothelial cells	Induction of the bone growth Angiogenesis Osteoblasts proliferation
Insulin-Like Growth Factor (IGF)	Osteoblasts Chondrocytes Endothelial cells	Osteoblasts proliferation ECM synthesis stimulate Osteoclasts proliferation

**Table 4:** In vitro vascularization strategies in bone tissue engineering.

Vascularization Strategy	Vascularization Technique
Use of the angiogenic GF (VEGF, PDGF, FGF)	Direct incorporation of the GF in the scaffold
Use of the angiogenic cell cultures	Direct delivery of endothelial cells into the implantation site (scaffold-based or scaffold free techniques)
Hypoxia-induced vascularization	Promoting the proliferation and sprouting of endothelial cells Recruitment of pericytes Inducing the expression of proangiogenic factors
Use of microvascular adipose tissue fragments	Seeding of the microvascular isolates onto the scaffolds
3D-bioprinting	Combining angiogenic GF and angiogenic cells with 3D-printing techniques (Laser-based methods, Extrusion printing)

## Data Availability

Not applicable.
